# GreekLex 2: A comprehensive lexical database with part-of-speech, syllabic, phonological, and stress information

**DOI:** 10.1371/journal.pone.0172493

**Published:** 2017-02-23

**Authors:** Antonios Kyparissiadis, Walter J. B. van Heuven, Nicola J. Pitchford, Timothy Ledgeway

**Affiliations:** School of Psychology, University of Nottingham, Nottingham, United Kingdom; University of Valencia, SPAIN

## Abstract

Databases containing lexical properties on any given orthography are crucial for psycholinguistic research. In the last ten years, a number of lexical databases have been developed for Greek. However, these lack important part-of-speech information. Furthermore, the need for alternative procedures for calculating syllabic measurements and stress information, as well as combination of several metrics to investigate linguistic properties of the Greek language are highlighted. To address these issues, we present a new extensive lexical database of Modern Greek (GreekLex 2) with part-of-speech information for each word and accurate syllabification and orthographic information predictive of stress, as well as several measurements of word similarity and phonetic information. The addition of detailed statistical information about Greek part-of-speech, syllabification, and stress neighbourhood allowed novel analyses of stress distribution within different grammatical categories and syllabic lengths to be carried out. Results showed that the statistical preponderance of stress position on the pre-final syllable that is reported for Greek language is dependent upon grammatical category. Additionally, analyses showed that a proportion higher than 90% of the tokens in the database would be stressed correctly solely by relying on *stress neighbourhood* information. The database and the scripts for orthographic and phonological syllabification as well as phonetic transcription are available at http://www.psychology.nottingham.ac.uk/greeklex/.

## Introduction

Lexical databases are essential tools in psycholinguistic research as they provide experimenters with lexical properties to manipulate in experiments and investigate their effects. They also allow researchers to carefully select their experimental stimuli to control for extraneous variables that are outside the scope of their theoretical questions of interest yet are known to affect language processing. In Modern Greek (henceforth, Greek), three databases have been developed in the past decade to address the apparent need for statistical information on Greek orthography. Two of them [1, 2] were developed from written corpora drawn from books and media sources. The third one is a corpus based on subtitle frequencies from films and television programs in Greek [3]. Furthermore, a database with word frequencies from online resources such as twitter, blog posts and newspaper web pages has been recently made available (WordLex, [4]). We first discuss the current databases and their limitations. Next, we present GreekLex 2, which includes new and improved grammatical, phonological, and orthographic information.

## Overview of psycholinguistic resources in Greek

### GreekLex

GreekLex [1] provided the first set of comprehensive orthographic information of a large set of Greek words. It was developed by compiling the entries of an orthographic dictionary (Dictionary of Standard Modern Greek, Aristotle University of Thessaloniki, 1998, as cited in [1]) and retrieving a number of statistical measurements for those entries from the Hellenic National Corpus (HNC, [5]), a collection of newspapers, books, periodicals and other written texts. The database comprises approximately 35,000 words that were present in both the dictionary and HNC. GreekLex includes only lemmas and not wordforms involving grammatical inflections. However, the database provides both joint lemma frequency and token frequency for each entry, with the latter representing frequency of the particular wordform that orthographically overlaps with the lemma (i.e. the frequency of observing, say, a particular verb in the first person, in singular, active voice and present tense). Ktori et al. [1] assessed the degree that GreekLex comprises a comparable sample to different versions of the overall HNC corpora (first version: 13 million entries [6]; second version: 43 million entries [7]). They showed that when plotting the summed word frequency in GreekLex as a function of word length, the (Poisson-like) distribution is very similar to those of the different HNC versions. They also found comparable average word-lengths (number of letters) and similar clustering of approximately 50% of the token frequencies around the four- and five-letter entries.

The lowercase version of GreekLex includes the length, lemma frequency and word (meaning, token) frequency measurements. The uppercase version of the database is stripped from double entries that in lowercase would constitute minimal stress pairs (e.g., *γέρος*, ´old´-*γερός*, ´strong´ become both *ΓΕΡΟΣ* in uppercase) because stress diacritics are not marked in uppercase Greek. Moreover, the uppercase version of GreekLex provides additional information such as orthographic neighbourhood (defined as the number of entries in the database that can be generated by replacing only one letter in the word of interest [8]), the number of orthographic neighbours that are of higher frequency, type and token bigram frequencies, and transposition, addition and deletion neighbours—i.e. the number of entries that only differ from the one of interest by i) two adjacent letters, ii) adding a single letter and iii) deleting a single letter, respectively. The database is freely available and the files can be downloaded at http://www.psychology.nottingham.ac.uk/GreekLex/.

### ILSP psycholinguistic resource

The ILSP Psycholinguistic Resource (IPLR, [2]) is a database that was also developed from the HNC corpus at the Institute of Language and Speech Processing (ILSP, Athens, Greece). The database’s size is approximately 217,000 entries and comprises all wordforms present in the corpus. IPLR offers abundant information on each entry such as syllabic measurements (number of syllables, stressed syllable, mean syllabic frequency), neighbourhood density (Cotlheart’s N measurement [8], Levenshtein distance [9], stress information, as well as sum and mean frequencies of the neighbours) and bigram sum and mean frequencies. These measurements are provided both considering types and tokens and both considering the stressed and non-stressed forms of the entries (that is, replacing the stressed vowel of multisyllabic entries with the corresponding one without a stress diacritic). Additionally, these measurements are provided using both the orthographic and phonological forms of each entry. Orthographic forms in the database were converted to phonetic transcriptions using a text-to-speech synthesis module [10] with high overall performance (>98%) followed by manual checking and corrections by the authors for ambiguous transcriptions. IPLR is available online (http://speech.ilsp.gr/iplr/) and includes various tools for searching quantitative and text information from the database or computing sublexical measurements for Greek words and nonwords not present in the database.

### SUBTLEX-GR

The third database that is available for research in the Greek language is based on counts derived from film and television subtitles. It was developed by Dimitropoulou et al. [3] and is available at http://www.bcbl.eu/databases/subtlex-gr/ from the Basque Centre on Cognition, Brain and Language (Donostia, Spain). SUBTLEX-GR was derived by compiling a corpus from Greek subtitle files and subsequently calculating frequency measurements from this corpus. It has been demonstrated in several languages (American-English [11]; British-English [12]; Dutch [13]; French [14]; Mandarin Chinese [15]) that subtitled-based frequencies account for a higher proportion of variance than traditional database frequencies in response times obtained from lexical decision and word naming tasks. Dimitropoulou and colleagues confirmed this for Greek as well and concluded that frequency counts and their corresponding relative values derived from a subtitle database seem better measurements and outperform the predictive capability of frequencies acquired from written sources.

SUBTLEX-GR consists of approximately 145,000 word-form entries encountered in the subtitle files. The variables provided involve frequency counts, relative frequencies, length, orthographic neighbourhood (Coltheart’s N) and Levenshtein distance. An additional measurement is contextual diversity, which indicates the number of different contexts (films or television series) in which each entry occurs. This variable has been shown, in some cases, to be a better predictor of reading performance than word frequency in behavioural tasks (e.g. [15]).

### Wordlex

Gimenes and New [4] recently made available a database with frequency measurements calculated from online resources, specifically twitter, blog posts and online newspapers. The database provides frequencies for 66 languages including Greek. Wordforms were crosschecked against a spellchecker. Comparisons made using behavioural data from lexical decision megastudies in several languages (not including Greek) showed that the new frequency measurements predicted reaction times equally well or sometimes better than existing frequencies (i.e. ‘traditional’ book-based, and subtitle frequencies). An advantage of this database is that the corpora where frequencies were calculated from are also available, hence allowing for additional calculations. However, at present, the database only provides word-frequency and contextual diversity measurements and no other statistical information on the word or sublexical level.

## Aims of GreekLex 2

Despite the considerable progress and effort in developing the databases reported above, certain psycholinguistic variables that impact language processing are currently absent from these psycholinguistic resources. A prominent variable is Part-of-Speech (PoS) information. In English, PoS category has been shown to affect reading behaviour in several studies. Baayen, Feldman and Schreiber [16] showed that monosyllabic verbs elicit faster reaction times than monosyllabic nouns in visual word recognition. Also, grammatical category correlates with stress position in English disyllables, with the typical pattern being nouns with pre-final stress and verbs with final stress. Participants have been reported to show sensitivity to this regularity of such words compared to their stress competitors in a variety of tasks [17, 18]. In a recent study with a similar rationale in Russian, adjectives (which are the only grammatical category that presents a statistical preponderance of stress on the first syllable) were shown to have a processing advantage in word recognition and naming when stressed on the regular position, which was not observed with nouns and verbs [19]. Hence, for certain designs, researchers might want to match their stimuli for PoS category. Additionally, function words, as opposed to content words, are usually avoided as experimental items in psycholinguistic research, while certain studies focus on specific PoS categories of words (e.g. [20]). These issues illustrate the apparent need of considering PoS information in various experimental designs and make it desirable to have this information readily available in psycholinguistic databases for experimental material selection.

The second point of concern with respect to Greek databases available so far is the syllabification of Greek words. This is relevant because there is evidence suggesting that syllables constitute independent units for reading, with the most typical phenomenon being the inhibitory effect of the initial syllable’s frequency in lexical decision [21–23]. Among the Greek databases, only IPLR provides information about syllabic units of Greek words. These units are based on phonological syllabifications whereas the orthographic syllabified entries provided are phoneme-to-grapheme conversions from the phonological forms. To extract the syllabic units in Greek, one needs to assign each fully pronounced vowel in the word to a separate syllable. In IPLR, syllabification is performed with the phonetic wordforms according to the principle of maximal onset [24], which posits that intervocalic consonants should be preferentially assigned to the subsequent syllable’s onset rather than the preceding syllable’s coda. Accordingly, syllabification was based on a list of phonologically acceptable initial syllabic consonant clusters based on the language’s phonotactics as proposed by Tzakosta and Karra [25]. When a consonant cluster was not in the list, the left part of the cluster was assigned to the coda of the previous syllable up to the letter that would result in the remaining part being an acceptable initial cluster. This was assigned to the following syllable’s onset. However, this approach results in some syllable-initial consonant clusters of which the phonotactic legality has been challenged by recent behavioural findings (e.g. ά-νθρω-πος ['a-nθro-pos], κα-μβάς [ka-'mvas]). Chaida, Gioulaki, Logotheti and Neocleous [26] investigated the tonal alignment of the stressed syllable in words with a two-consonant cluster before the stressed vowel. They found that, in word production, whether the first consonant of the cluster was syllabified with the coda of the preceding syllable or the onset of the following one depended on whether the two consonants constituted a legal initial syllable cluster or not. Consonant clusters like [mv] in the above example were split to the previous coda and following onset respectively. We calculated the proportion of such cases, by analysing the approximately 500 consonant clusters in IPLR and GreekLex and found that parsing according to the above approach resulted in 64% (320) syllable-initial clusters that fall within this category. Importantly, such clusters appear in 16% of the GreekLex entries. Therefore, an alternative syllabication method is also provided for such consonant clusters.

A third area that warrants further investigations and necessitates statistical information is stress position and its relationship with lexical and sublexical information. Research involving corpus analyses of several languages including Greek (e.g. [27, 28]) has revealed that the orthography provides information about stress position through statistical regularities of the language, even beyond explicitly indicating this position by stress diacritics. Behavioural findings suggest that readers are sensitive to subtle statistical information that might emerge only from sub-groups of words in the mental representations. For example, stress neighbourhood involves the consistency of each specific word ending regarding the syllabic position of stress. Experimental work conducted in Italian [29, 30] showed that Italian readers are sensitive to stress neighbourhood when assigning lexical stress. Furthermore, Burani and Arduino [30] found that words with many stress neighbours (defined in terms of syllabic stress position within the group of words that share the same ending) are named faster than those with many stress competitors. They also argued that stress neighbourhood is a more prominent factor for assigning stress in Italian than stress regularity. The latter refers to the default metrical pattern, a statistical predominance of a specific syllabic position to receive stress more often than the other syllables, which has been shown to affect the stress assignment mechanism in several languages (English [31], Italian [29], Greek [32], Dutch [33]). Therefore, it was essential to provide such information in GreekLex 2.

The inclusion of the above described variables in GreekLex 2 made it possible to conduct a series of new analyses regarding the predictability of stress position based on different information, such as PoS, as well as the orthographic and phonological endings of words. Furthermore, making available scripts that implement the algorithms for phonetic transcription and orthographic and phonological syllabification will allow other databases lacking these variables to be enhanced with this information. Finally, several important additional variables were also included in GreekLex 2: Zipf values [12], OLD20 [9], which is an improvement of the traditional orthographic similarity measure Coltheart’s N [8], and phonological information on the entries.

The variables available in GreekLex 1 were based on lemmas. An important question is whether lemmas constitute a representative sample of a given language to calculate sublexical and lexical measures because the frequency of statistical units might be over- or under-estimated ([34]). This is particularly important for inflectional languages such as Greek because mainly the suffixes of wordforms present a high level of variability that signifies changes in several grammatical characteristics of the word (e.g., for verbs: person, number, tense, conjugation, voice etc.; for nouns: case, number). This issue has been addressed in GreekLex 2 because the measurements included in the database have been calculated from the underlying wordforms. Calculations based on the lemma forms only are also provided allowing future investigations regarding the degree of similarity between the metrics derived from the two sources and whether those calculated from lemma forms are accurate measurements of the language’s statistical characteristics. The following section describes in detail the new information added to GreekLex 2 and presents descriptive information about the new variables.

## GreekLex 2

Below we summarize the new information added to GreekLex 2: part-of-speech information, syllabic information, phonetic transcriptions, stress neighbourhood measurements, Zipf values, and Levenshtein distance-based measurements of orthographic similarity. As all the new information provided was calculated by considering the stressed and unstressed versions of the entries, the uppercase version of the GreekLex 1 database was deemed redundant and was not further updated.

### Part-of-Speech (PoS) information

Part-of-speech information is provided for each entry in GreekLex 2. The typical procedure of adding part-of-speech information in a lexical database requires the original texts that the corpus was built from and applying a statistically trained PoS tagger to annotate all the tokens in the corpus (e.g. [35]). As the texts of the HNC that GreekLex was generated from were not freely available, it was impossible to follow this procedure to add PoS tags to the entries of the database. However, GreekLex only contains lemmas, which allowed cross-checking each entry against a dictionary containing such information and acquiring all the possible PoS categories from each lemma. We followed this approach and cross-checked the entries of the database against the Dictionary of Standard Modern Greek [36] which is available online at http://www.greek-language.gr/greekLang/modern_greek/tools/lexica/triantafyllides/index.html.

The most frequent PoS type in GreekLex 2 was Noun (58.5%), followed by Adjective (25.8%) and Verb (14.2%). Only 1.7% of the entries were Adverbs and the remainder of the categories had frequencies lower than 1% (e.g., Articles, Quantifiers, Prepositions, etc.). Entries that could be characterised as problematic were those that were orthographically identical yet multiple inflections of the same lemma. For example, the form *επάξια* could be the adverb of the adjective *επάξιος*, the feminine adjective in singular and nominative case, or the neutral adjective in plural in either nominative or accusative case. Although it is clear that such entries were initially included in the lemma database representing the adverbial form which is typically present in dictionaries, the additional, word-form PoS categories could not be ignored and were added as secondary tags. Information about the tag abbreviations used in the database and descriptive statistics of the frequency of each PoS category are provided with GreekLex 2.

To evaluate if our automated PoS tagging method returned reliable results, three linguists who had no access to the annotated data were asked to manually annotate a proportion of the database’s entries. 1000 items (approximately 3%) were manually annotated by all three linguists. The levels of agreement with the automated method were 98.5%, 97.6% and 98.3%. The inter-rater reliability was 95.8% (*k* = 0.958, *z* = 72.2, *p* < .001, N = 999) based on Fleiss’ kappa [37] adaptation of the Cohen’s kappa statistic for multiple raters and categorical data. Items with inconsistent tags among the linguists or between them and the automated method were mostly nominalised adjectives such as *μασκοφόρος* (m. *masked man*) [noun] *vs* [adj], and *τραχεία* (m. *trachea* but also *rough*-feminine gender) [adj] *vs* [noun]. Nonetheless, such cases highlight the ambiguity that arises in this task because it is possible to assign more than one PoS category to Greek words, even in their lemma form. Consequently, researchers using the derived PoS information need to be aware that multiple tags could be assigned to several entries in the database. In the future, databases that include PoS information should use the text corpora that they are generated from to disambiguate PoS categories for ambiguous entries using contextual information.

### Syllabic units

Syllabic information, including the syllabic units each entry consists of, the number of syllables, the stressed syllable of polysyllabic entries and the mean syllabic frequency when considering or ignoring the stress marks has been included in GreekLex 2. Type and token syllable frequencies are provided separately because there are indications that the two might have different effects in visual word recognition [38]. The counts and standardised frequency of occurrence per million for the syllabic units are available in separate files.

Orthographic syllabification was performed according to the rules presented in the *Modern Grammar of Demotiki* (m. Modern Greek) [39], which is formally taught in school and is applied when writing in order to split the words that reach the end of the line and need to be continued onto the next one. The rules posit that a cluster can constitute the beginning of a syllable if there is a Greek word that starts with such a cluster. Otherwise, the first letter goes with the previous syllable and the remaining consonant(s) are assigned to the beginning of the subsequent syllable. To develop a list with all legal initial consonant clusters, all entries from the three currently available databases in Greek were considered. GreekLex 2 is the first database that provides orthographic syllabic units based on syllabification rules of Greek school grammar. However, whether these units are relevant in Greek reading requires further research.

**Fig 1:**
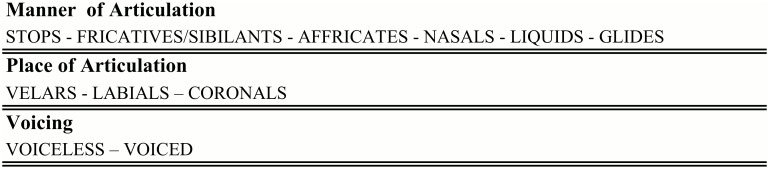
Consonant-type classification. Consonant types were classified according to the Manner of Articulation, Place of Articulation, and Voicing scales.

In terms of parsing the phonological syllables, the maximal onset principle was applied on the basis of phonotactically acceptable consonant combinations. Tzakosta and Karra [25] proposed a two-dimensional scale involving manner (MoA) and place (PoA) of articulation when the left consonant is a member of a leftmost consonant category compared to the category on the right on at least one of these dimensions (classification of consonant types for MoA and PoA are presented in Fig 1). The above procedure was also applied in IPLR but certain entries were re-evaluated in the way consonant clusters are separated between adjacent syllables. Such cases mostly involve clusters with nasal ([m], [n], [ɲ]) and liquid ([l], [r], [ʎ]) consonants, which according to Tzakosta and Karra [25], are only evaluated on the PoA dimension due to their unclear theoretical status with respect to MoA. There are two issues related to this. The first is that such clusters seem to be given a ‘free pass’ and considered ‘legal’ without evaluation on the PoA dimension, because otherwise clusters like [rm] (e.g. *άρμα*) and [rp] (e.g. *άρπα*) would still be considered ‘illegal’, in contrast to IPLR’s output (['a-rma], ['a-rpa]). The second issue involves clusters with such consonants that are correctly considered acceptable when only considering PoA, yet word production data [26] reported earlier are inconsistent with this approach. Therefore, to offer an alternative approach, a less liberal methodology was followed in GreekLex 2 and clusters containing the previously-described consonant combinations were still evaluated both in MoA and PoA, in which case entries such as *κα**μβ**άς* [ka'mvas] have the consonant cluster split. Additionally, an extra dimension was introduced as a criterion that involves a binary voicing scale (see Tzakosta [40]) that is adequate on its own to force a cluster split (also presented in Fig 1). Furthermore, an additional constraint was set regarding initial liquid or nasal-liquid clusters which were always treated as heterosyllabic. Finally, a small number of clusters retrieved from the set provided by Botinis [41] which would otherwise be treated as heterosyllabic by the algorithm are added as legal ones. These rules resulted in a novel set of acceptable clusters which is included in GreekLex 2.

A python script that implements the algorithm based on the rules described above was developed by the first author and is provided together with the database. As mentioned earlier, of the approximately 500 initial consonant clusters considered legal in IPLR and handled as tautosyllabic, 64% (320) were assessed as ‘illegal’ according to the improved syllabification algorithm. This apparently high proportion was mainly due to clusters with more than two consonants (240 out of 320). These were subsequently parsed with the legal onset criterion that aimed to maximise the number of consonants on the onset to the level they would constitute a legal cluster, but cannot guarantee the legality of codas. Applying this approach had an impact on the phonological syllabification of approximately 16% of types and 5% of tokens in the database. The different approaches followed in the orthographic and phonological syllable parsing mean that the orthographic and phonological syllabic units in GreekLex 2 do not always overlap. The phonological syllables were converted back to the orthographic code as well and are also included in the database. Consequently, there are two forms of orthographic syllabic units; those emerging from traditional grammar rules which were previously unavailable from other psycholinguistic resources, and those emerging from phonology.

An additional issue in terms of syllabification are wordforms that contain an ambiguous CVV combination. In these cases, the orthography alone cannot determine whether the middle vowel is fully pronounced or not. When it is pronounced, such letter clusters are parsed into two separate syllables whereas when it is not, they comprise one syllable. The most frequent ambiguous CVV combination is the CiV pattern (see [42]) in which *i* represents different vowels (e.g., *ι*, *η*, *υ*) or vowel combinations (e.g., *ει*) that, in isolation, correspond to the [i] sound. In such CiV cases, however, the vowel sound is either fully realised into [i], resulting in two syllables (*κυά**νιο* [ci-'a-ni-ο]), or not, resulting in one syllable [*γυά**λα* ['ʝa-la]]. Approximately 16% of the entries in GreekLex 2 contained such ambiguities. A simple possible solution that was explored is to consider all words containing each cluster and determine the percentages of such vowel combinations being parsed into one and two syllables. Then all instances of a given cluster can be parsed according to the majority of cases calculated for this specific cluster. For example, the vowel cluster *ιο* is parsed into separate syllables at a rate higher than 80% of cases hence all instances of this cluster were parsed into two syllables. Despite the fact that this approach guarantees that there will be errors in the instances of ambiguous clusters that are not parsed consistently with the majority of cases, this method resulted in an overall accuracy of 95%. Only 71% of the ambiguous clusters were parsed correctly when applying this approach. Therefore, all entries containing ambiguous entries were cross-checked and the syllabification of erroneous ones was corrected manually. For ambiguous cases, where both pronunciations were acceptable (e.g. *διάφανος* [δi-'a-fa-nos], ['δʝa-fa-nos]), an orthographic dictionary with syllabic information [43] was consulted for purposes of consistency. A similar approach was also used for IPLR because CiV cases were submitted to manual verification [42]. However, despite the high accuracy of the automatic approach in conjunction to the manual inspection, errors can naturally not be avoided (e.g. *διαβάτης* GreekLex 2: [δʝa-'va-tis], IPLR:[δi-a-'va-tis]; *κλεψιά* GreekLex 2: [kle-'psça], IPLR:[kle-psi-'a]). A comparison between the two databases in relation to this issue revealed inconsistent transcriptions with regards to the CiV pattern (the comparison file is provided in GreekLex 2). However, it should be noted that very often such discrepancies do not reflect an error in the transcription available in either of the databases but rather alternative transcriptions that are both correct. Nonetheless, GreekLex 2 adheres to the dictionary information to ensure a consistent approach is adopted for all words in the database. Table 1 provides descriptive statistics for the distribution of types and tokens by their syllabic length.

**Table 1:** Counts and proportions (percent) of type and token distribution by syllabic length.

	Types		Tokens	
Syllabic length	Counts	Percentage	Counts per million	Percentage
1	276	0.8	225186	53.5
2	3661	10.4	100472	23.9
3	8391	23.8	43699	10.4
4	10645	30.2	33264	7.9
5	7822	22.2	13783	3.3
6	3270	9.3	4169	1
7	921	2.6	453	0.1
8	252	0.7	128	0
9	54	0.2	50	0
10	11	0	10	0
11	1	0	3	0

### Phonetic transcriptions

Phonetic transcriptions of the orthographic strings in GreekLex 2 were generated with an algorithm implemented in Python capitalising on the high level of grapho-phonemic consistency of Greek orthography at least in the feedforward direction, that is, from print to sound [42]. However, because these are computer generated transcriptions, they can only be an approximation to Greek oral speech. Ambiguous CVV cases were handled by utilising syllabic information generated in the previous phase of processing as described in the prior section and more precisely whether the two potential vowel graphemes were in the same syllabic unit or not. When they are tautosyllabic, the medial vowel grapheme is fully pronounced (*αγο**νιώ* [a-γο-ni-'ο]), and when they are heterosyllabic, the grapheme is not realised as a vowel and, in the case of the previous consonant being [n] or [l], it is palatalised and converted to [ɲ] and [ʎ] respectively (*νιώ**θω* ['ɲο-θo]). However, this approach does not imply or reflect a relationship between orthographic syllabification and the phonetic transcriptions emerging in cases of palatalisation as this is a theoretical issue pertinent to the phonological domain that is not trivial to universally resolve.

To assess how well our algorithm performed in this task, we compared the common entries between GreekLex 2 and IPLR since the latter was phonetically converted using a highly accurate text-to-speech module. This resulted in 24,627 common entries between the two databases. The only changes made before the comparison were: i) The phonetic transcription of some monosyllabic entries in IPLR had the syllable’s vowel stressed; this was assessed as redundant and replaced with the unstressed version of the same vowel. ii). Protopapas et al. [2] indicated that in IPLR all cases of nasal consonants followed by homorganic stops (e.g. [mb],[nd]) were simplified by dropping the nasal, an approach which was also followed in our phonetic conversion algorithm. However, we observed that in the downloadable version of IPLR that was available at the time of our analyses (21/09/2016, ‘all_num_clean_text.txt’), there was variation with respect to whether the nasal was dropped or not (e.g. *ακουμπάω* [aku'mbao], *ακουμπήσαμε* [aku'bisamε]). Consequently, this simplification was applied to all entries in IPLR containing such combinations by removing the nasal. iii) For the exact same reasons, the labiodental nasal [ɱ] is treated as identical to the bilabial nasal [m].

The level of agreement in phonetic transcriptions of the common entries between our algorithm and the procedure applied in IPLR was 98%. This is notable because it shows the level of feedforward transparency of Greek orthography. The most substantial inconsistency of Greek orthography involving the CVV pattern was overcome by providing this information to the algorithm. Hence, the set of hand-written rules included in the current algorithm was almost equally efficiently as a rule-based algorithm [10] trained on an excessive amount of data (approximately 900,000 words). A qualitative investigation of the inconsistent transcriptions revealed that the vast majority of them involved entries where the CiV pattern could produce acceptable pronunciations either when [i] was fully pronounced or not (e.g. *διάφανος* [δi'afanos], ['δʝafanos]). Remaining inconsistencies mainly involved differences in pronunciation that could be broadly classified as allophones (e.g. *αντένα* [a'dεna], [a'ntεna]; *ελέγκτρια* [ε'lεŋgtria], [ε'lεgtria]).

The algorithms for performing the tasks of syllabification and phonetic conversion are implemented in Python. The scripts are useful for processing large databases and are freely available online in contrast to similar software for Greek that have been typically developed for commercial purposes or restricted use (e.g. [10, 44]) and cannot be used for large datasets.

### Measurements of stress regularity

Two distinct features have been added in GreekLex 2 regarding the word neighbourhoods that emerge from stress position and word endings. For reasons of disambiguation, one is referred to as *Rime Neighbourhood* and the other as *Stress Neighbourhood*. Rime Neighbourhood refers to the number of entries in the database that match the target entry from the stressed vowel up to the final letter of the word. This metric can be also obtained from IPLR’s online search tool. Stress Neighbourhood (as proposed by Colombo [29]) involves the orthographic ending of each entry, comprising the nucleus vowel of the pre-final syllable up to the final letter of the word. Stress diacritics are omitted up to this point of processing. Then the metric is generated after calculating the proportion of entries having stress on the same syllabic position as the entry of interest over all entries sharing the same ending with it. For both Rime and Stress Neighbourhood and their corresponding variables, type and token counts as well as calculations on the orthographic and phonological strings are provided separately. The average rime length of the polysyllabic entries in the database was 3.7 letters (*SD* 1.9) and the average final sequence length based on Colombo’s criterion was 3.6 letters (*SD* 0.9).

In a comparative study involving corpus analyses in several languages, Monaghan, Arciuli and Seva [28] showed that in Greek, both beginning and ending letters convey cues regarding stress position and depending on the word’s number of syllables they can have high predictive value, even though stress position in Greek is explicitly marked with diacritics in lowercase. In a quantitative analysis, we assigned each polysyllabic entry in the database with stress at the position that was most frequent among the entries it shared a common orthographic ending or rime with. When considering the orthographic endings according to Colombo’s approach, we found that 86.9% of the types and 91.3% of tokens in the database would be stressed correctly by following this simple procedure. Repeating the same procedure with the rimes was less straight-forward given that each entry’s rime varied in length according to stress position and there were many overlapping endings matching the same entry. The approach of hierarchising endings from longer to shorter ones resulted in 75.5% accuracy for types and 80.0% for tokens.

Hence, it seems that Greek orthography provides sublexical information on stress position that could be utilised by the reader to assign stress. Also, stress neighbourhood seems to offer more straightforward information than rime neighbourhood in terms of stress position. It has been shown that the stress assignment mechanism in Greek is a much more complex process than to simply decode the orthographic diacritics to determine stress position. Rather, it also involves lexical and statistical information [32]. Grimani and Protopapas [45] used pseudowords and found significantly larger effects of their suffixes on stress assignment when the suffix did not have any stress competitors compared to when it did, thus showing that such effects originate in the distributional properties of the language. Therefore, it remains to be further investigated empirically whether the measurements presented here constitute sources of stress information that operate in Greek reading.

So far, the focus was on statistical characteristics of stress distribution involving subgroups of the lexicon (e.g. words ending in a given letter string); next, we focus on the default metrical pattern. Naturally, the preponderance of words receiving stress on a given syllabic position does not have consistent statistical characteristics across all languages. For example, stress assignment of Italian polysyllabic words with more than two syllables is the only instance of print-to-sound ambiguity in Italian. But approximately 80% of these words are stressed on the pre-final syllable (Thornton, Iacobini, & Burani, as cited in [30]) and these words were indeed shown to be read and recognised faster and/or more accurately than those stressed on the other positions [29]. Similarly, more than 80% of English disyllabic words in the CELEX database [46] receive stress on the pre-final syllable and this seems to generate similar experimental findings with Italian [31] (but see [47]). In Greek, Protopapas [48] calculated that monosyllables take up approximately 38% of all words, and for polysyllables the stress position distribution is as follows, Final: 19%, Pre-final: 28%, Antepenultimate: 16%. As illustrated in Table 2, the token analysis of the lemmas in GreekLex 2.0 presents a similar distribution.

**Table 2:** Counts and proportions (percent) of type and token distribution of stress position.

	Types		Tokens	
Stress position	Counts	Percentage	Counts per million	Percentage
Monosyllables	276	0.8	225192	53.2
Final	9951	28.2	61558	14.6
Pre-final	13569	38.4	88817	21
Antepenultimate	11508	32.6	47368	11.2

**Note**. Monosyllables are presented separately in the analysis as they can only be stressed on their sole syllable.

**Fig 2:**
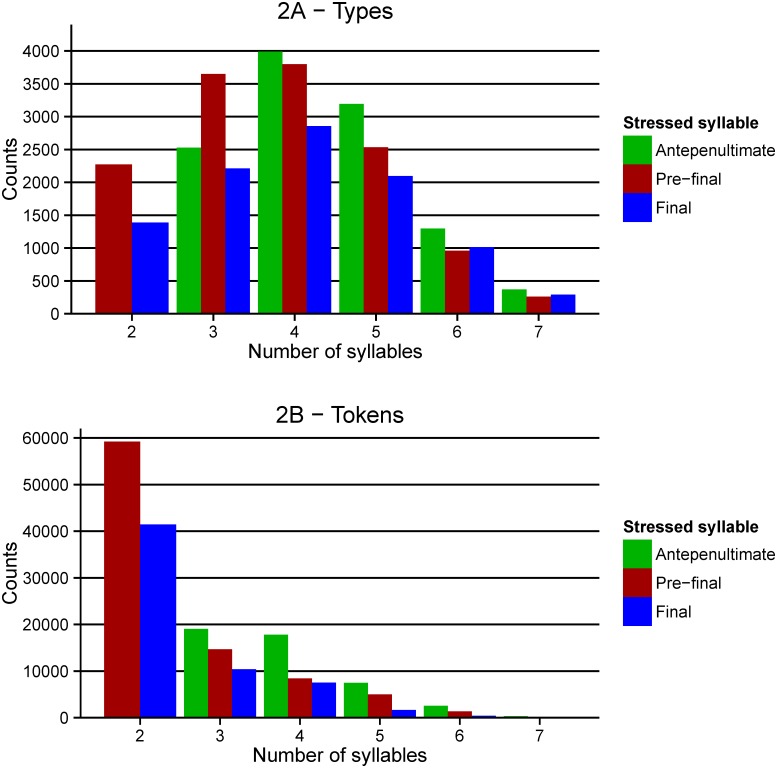
Distribution of stress position by number of syllables. Distribution of stress position by types (2A) and tokens (2B). Syllabic lengths higher than 7 (up to 11) were not presented in the graph as they accumulatively represent a proportion less than 1% (types) and 0.1% (tokens) of the whole set.

As illustrated in Table 2, the pre-final position only presents a relative dominance as it is higher only when compared to each of the other positions separately. It is not the most frequent in comparison to the other two positions accumulatively. Given that the research discussed above seems to focus on the default pattern and specifically on subsets where the dominance is observed (e.g. disyllables in English), the distribution of stress position was calculated separately for each different syllabic length occurring in the database (see Fig 2).

Interestingly, Fig 2 illustrates that only disyllabic words consistently show the statistical dominance of the pre-final in both the type and token analysis, and trisyllabic words only in the type analysis. All the other syllabic lengths show a statistical dominance of stress on the antepenultimate syllable. This is important because the default pattern has been shown to be an active source of stress information in behavioural experiments with items larger than disyllabic ones (e.g. Protopapas, Gerakaki, & Alexandri [49] used items of 3–5 syllables). Hence, the metrical pattern seems to be active even with sub-groups of words that, when seen in isolation, do not present this statistical dominance of the pre-final syllable. This could perhaps be attributed to the highest overall number of disyllabic token items (see Fig 2B) compared to the other polysyllabic words. A potential concern is that this analysis is based on lemmas and not on wordforms. This is particularly important because in Greek there are several cases in which inflected forms have a different stress position than their lemma (see [49]). To verify whether the same patterns would emerge with wordforms as well as from a different source, we repeated the analysis with IPLR’s entries. Results showed that the patterns were very similar to the lemma entries of GreekLex 2 (correlation for types: *r* = .95, *p* < .001; for tokens: *r* = .98, *p* < .001; the tables can be found in the GreekLex 2 materials). Importantly, the crucial observation of non-uniform statistical predominance of stress on the penultimate syllable was found in GreekLex 2 and IPLR.

**Fig 3:**
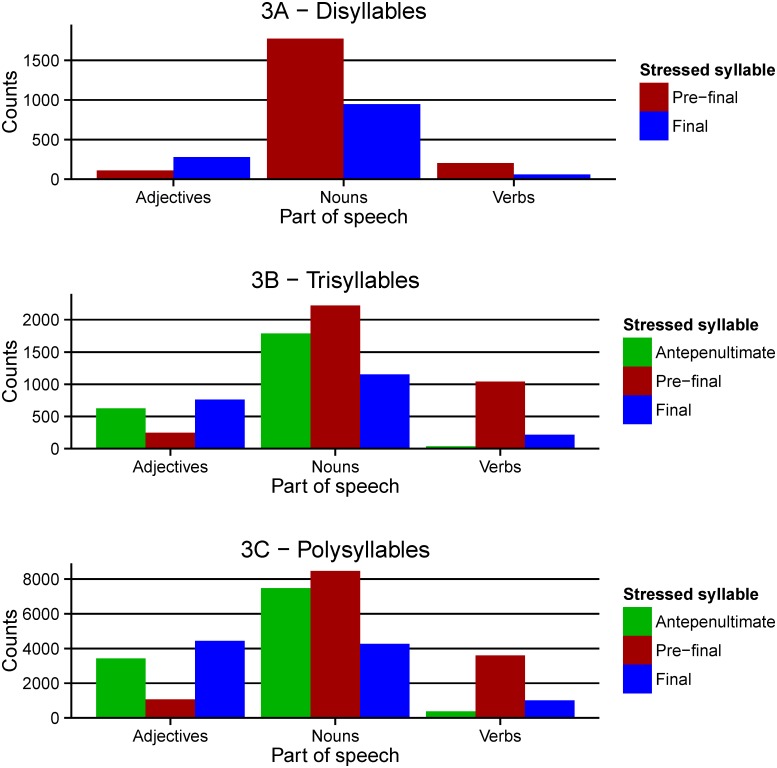
Distribution of stress position by part-of-speech category. Counts for disyllables (3A), trisyllables (3B), and all polysyllables (3C). Only adjectives, nouns and verbs were considered for these calculations.

Finally, the availability of PoS, syllabic, and stress information makes it possible to investigate whether the Greek language has any regularities with respect to grammatical category and stress position. In English, disyllabic nouns typically present a trochaic stress pattern while disyllabic verbs typically present an iambic stress pattern and this has been shown to affect reading behaviour in several psycholinguistic tasks [17–18]. Analysis with GreekLex 2 involved the three major PoS categories of nouns, adjectives and verbs that take up 98% of all the entries in the database. As illustrated in Fig 3, nouns and verbs are broadly following the overall pattern in all analyses of disyllabic, trisyllabic and polysyllabic stimuli. Interestingly, adjectives do not follow this pattern. In sharp contrast to the default bias towards the pre-final syllable, in the case of adjectives this syllabic position is actually disfavoured. However, it should be noted that this analysis is only based on lemmas and it remains to be confirmed whether the same pattern would emerge if wordforms were considered. Unfortunately, none of the Greek databases containing wordforms have PoS information, hence this analysis cannot be currently performed. However, the emergence of similar patterns when considering lemmas (Greeklex 2) and wordforms (IPLR) in the analysis of stress position in relation to syllabic length suggests that similar patterns for lemmas and wordforms could also be expected for PoS.

### Measurements of orthographic similarity

As an alternative to Coltheart’s N (i.e. the number of words in a database that have the same length and only differ in one letter from a given entry [8]), Levenshtein distance [9] has recently been gaining ground as a more sensitive metric of orthographic similarity because it discerns differences between orthographic strings that would not be captured by Coltheart’s definition of neighbourhood. It is defined as the number of insertions, substitutions and deletions required to produce an orthographic string from another string. OLD20 [9], which is provided in GreekLex 2, is the mean Levenshtein distance for each word in order to generate its 20 closest orthographically similar words. While a high N density shows that there are many other words in the database that are only one letter away from a given word, it is a low OLD20 that indicates large orthographic overlap with the 20 closest items in the database. These measurements were calculated both when considering the stressed and the unstressed versions of the entries. Furthermore, they were calculated by considering only the lemmas, which the database currently consists of, and also by considering all the wordforms from the HNC corpus that are assigned to each lemma.

**Fig 4:**
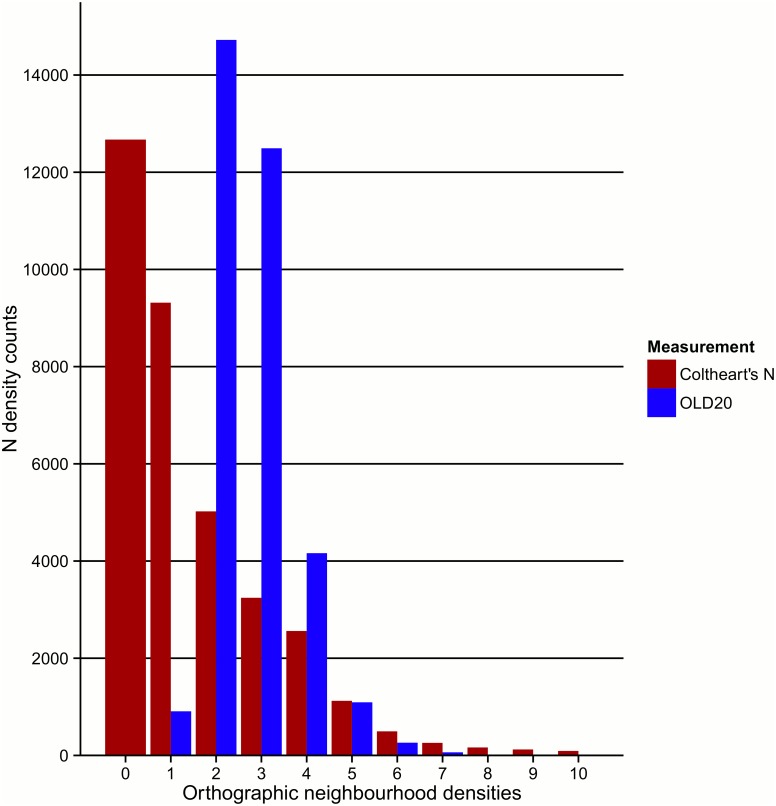
Frequency of N (Neighbourhood) counts. Frequency counts for the clustered OLD20 Levenshtein Distance (based on [9]) values and Coltheart’s N orthographic similarity (based on [8]) values. OLD20 values are clustered around their closest integer numbers (e.g. a value of 2 represents the counts of all values between 1.5 and 2.49). Coltheart’s N values above 10 are not presented in the graph as they accumulatively represent a proportion smaller than 0.5% of the whole set.

**Fig 5:**
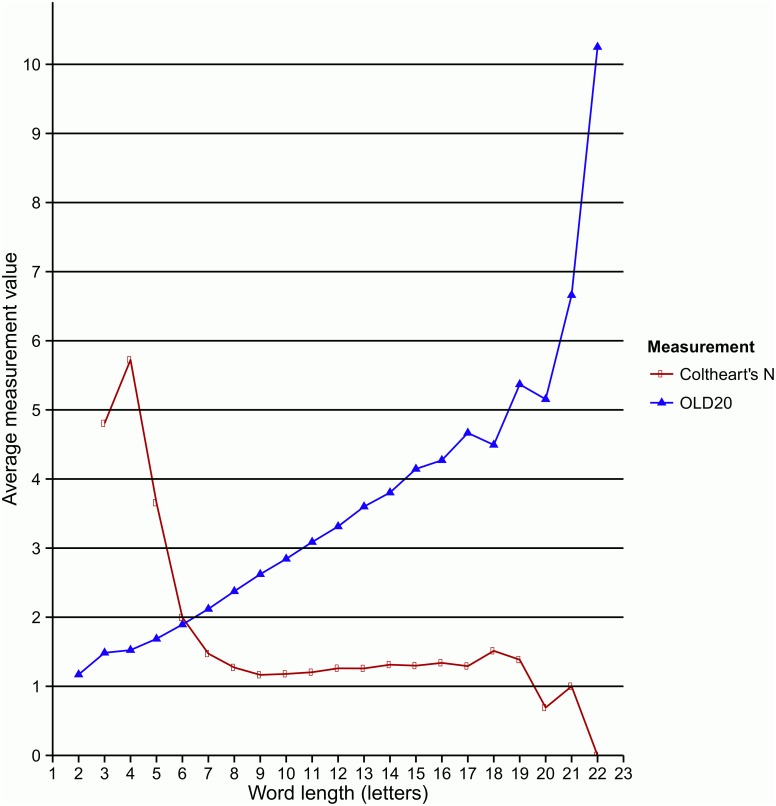
Orthographic similarity as a function of length. Distributions of mean OLD20 Levenshtein Distance (based on [9]) and Coltheart’s N orthographic similarity (based on [8]) as a function of word length measured in letters.

As shown in Fig 4, the Coltheart’s N density counts that are by far the most frequent in the database are zeros (i.e. no orthographic neighbours). The most frequent OLD20 values cluster around the value of 2 (1.5–2.49). The figure illustrates the counts of both densities (N) and averaged similarity values (OLD20) when considering all wordforms for neighbours rather than just the database’s lemmas. As expected and illustrated in Fig 5, Coltheart’s N density depends upon word length, with shorter words overall presenting higher densities. Similar patterns have been observed and reported by Ktori et al. [1] in an analysis that only considered lemmas as potential neighbours and only those with a frequency higher than 1 per million. A comparable pattern was observed when computing the OLD20 measurement. In this case, higher values indicate lower amount of orthographic overlap with their closest entries, hence the adverse pattern is observed. Again, Fig 5 also depicts the patterns emerging after considering all wordforms related to the database’s lemmas. Finally, these measurements were also calculated for the phonetic forms of the entries (PLD20, both the stressed and unstressed versions). As expected, due to the high level of grapho-phonemic consistency of the Greek language, these indices highly correlated with their corresponding orthographic measures. Significant correlations were found between the orthographic and phonological Coltheart’s N measurements for stressed (*r* = 0.79) and unstressed forms (*r* = 0.79), as well as between the OLD20 and PLD20 for both stressed (*r* = 0.95) and unstressed (*r* = 0.96) versions of the entries (in all cases, *n* = 35283 and *p* < .001).

### Zipf values

Log transformed word frequency values based on the Zipf scale are now provided in conjunction with the traditional measurement of frequency per million (fpmw). As pointed out by van Heuven et al. [12], there is a relatively large proportion of entries in large corpora that have a frequency lower than 1 fpmw and the word frequency effect is strongest for words below 1 fpmw. The problem with frequencies below 1 fpmw is that these become negative when a logarithmic transformation is applied. Therefore, van Heuven et al. [12] proposed the Zipf scale of word frequency, which has a number of properties that make it easier to understand. In particular, it is a logarithmic scale with a few points and the middle of the scale separates low from high frequency words. The Zipf values for the entries in GreekLex 2 are calculated using the formula proposed by van Heuven et al. [12]:

$$Zipf=log10\left(\frac{frequency\;count+1}{46.89+0.0353}\right)+3.0$$ (1)

where the first number of the denominator represents the corpus size and the second one represents the number of unique word types in the database, both measured in millions.

## Summary and future development

We have presented GreekLex 2, an updated version of the GreekLex database [1], reporting information such as part-of-speech category, which is not available in other Greek databases and alternative information about syllabic units and orthographic measurements that provide predictability on stress position. We first presented a summary of the first version of GreekLex as well as other databases available in Greek and highlighted areas for further development. We reported the procedures followed to generate the new information. The database now provides part-of-speech information for each entry; accurate orthographic and phonological syllabification as well as syllabic length and stress position; *stress* and *rime neighbourhood* measurements that indicate the regularity of stress position for each entry based on its ending. Additionally, we presented novel analyses that the availability of the new features allowed and investigated the regularities that Greek language presents. We showed that the distribution of stress position does not uniformly adhere to the pre-final syllable bias but is dependent upon grammatical category. Additionally, we showed that a proportion higher than 85% of the entries in the database would be stressed correctly solely by relying on *stress neighbourhood* information. Plans for future developments of the database include adding information that is less dependent upon computational and lexicographical tools like phonetic transcription modules or dictionaries, and more reliance on naturally-produced speech (to generate phonetic transcriptions) and intact text corpora (to generate syntactic information directly from such sources).

The database is provided in text files encoded in ASCII and UTF-8 (for users that do not have Greek installed on their system) and as a comma-separated values (csv) file to be opened with spreadsheet software. All files which are related to this publication, such as the database, the complementary files with syllabic frequencies, Stress Neighbourhood and PoS information, and the Python scripts for automatic syllabification and phonetic transcription can be found at http://www.psychology.nottingham.ac.uk/greeklex/ and https://github.com/CypressA/GreekLex-2. An overview of these files and the information each provides is presented in the supplementary materials.

## Supporting information

S1 File Supporting information. (DOCX)
