# Supplementary material for: GreekLex 2: A comprehensive lexical database with part-of-speech, syllabic, phonological, and stress information
Source: PLoS One. 2017 Feb 23;12(2):e0172493. doi: 10.1371/journal.pone.0172493 (PMC5322960; doi:10.1371/journal.pone.0172493)
Supplement: S1 File — (DOCX) [file pone.0172493.s001.docx]

Tables presenting the information provided by each file in GreekLex 2.

**Table 1. The GreekLex 2 database.**

| Column | Name | Property |
| --- | --- | --- |
|  |  |  |
| 1 | Word | The actual word in lowercase |
| 2 | Length | Number of letters in the word |
| 3 | LemmaFreq | HNC* lemma written frequency per million |
| 4 | WordFreq | HNC* wordform written frequency per million |
| 5 | zipfValue | Zipf value based on the frequency count |
| 6 | Syllables | Orthographic syllabic units comprising each entry |
| 7 | SyllLength | Number of syllables |
| 8 | StressPosition | Syllabic position of stress relative to the final syllable |
| 9 | MeanSylFreq(Stress-types-lemmas) | Mean orthographic syllabic frequency with stress diacritics based on lemma type frequencies |
| 10 | MeanSylFreq(Stress-tokens-lemmas) | Mean orthographic syllabic frequency with stress diacritics based on lemma token frequencies |
| 11 | MeanSylFreq(noStress-types-lemmas) | Mean orthographic syllabic frequency ignoring stress diacritics based on lemma type frequencies |
| 12 | MeanSylFreq(noStress-tokens-lemmas) | Mean orthographic syllabic frequency ignoring stress diacritics based on lemma token frequencies |
| 13 | MeanSylFreq(Stress-types-wordforms) | Mean orthographic syllabic frequency with stress diacritics based on wordform type frequencies |
| 14 | MeanSylFreq(Stress-tokens-wordforms) | Mean orthographic syllabic frequency with stress diacritics based on wordform token frequencies |
| 15 | MeanSylFreq(noStress-types-wordforms) | Mean orthographic syllabic frequency ignoring stress diacritics based on wordform type frequencies |
| 16 | MeanSylFreq(noStress-tokens-wordforms) | Mean orthographic syllabic frequency ignoring stress diacritics based on wordform token frequencies |
| 17 | PhonTranscription | Phonetic transcription of the orthographic form |
| 18 | PhonLength | Number of letters in the word |
| 19 | PhonSyllables | Phonological syllabic units comprising each entry |
| 20 | PhonSyllables(spelling) | Orthographic syllabic units comprising each entry resulting from phonological syllabification |
| 21 | MeanPhonSylFreq(Stress-types-lemmas) | Mean syllabic frequency with stress diacritics based on lemma type frequencies |
| 22 | MeanPhonSylFreq(Stress-tokens-lemmas) | Mean orthographic syllabic frequency with stress diacritics based on lemma token frequencies |
| 23 | MeanPhonSylFreq(noStress-types-lemmas) | Mean orthographic syllabic frequency ignoring stress diacritics based on lemma type frequencies |
| 24 | MeanPhonSylFreq(noStress-tokens-lemmas) | Mean orthographic syllabic frequency ignoring stress diacritics based on lemma token frequencies |
| 25 | MeanPhonSylFreq(Stress-types-wordforms) | Mean syllabic frequency with stress diacritics based on wordform type frequencies |
| 26 | MeanPhonSylFreq(Stress-tokens-wordforms) | Mean orthographic syllabic frequency with stress diacritics based on wordform token frequencies |
| 27 | MeanPhonSylFreq(noStress-types-wordforms) | Mean orthographic syllabic frequency ignoring stress diacritics based on wordform type frequencies |
| 28 | MeanPhonSylFreq(noStress-tokens-wordforms) | Mean orthographic syllabic frequency ignoring stress diacritics based on wordform token frequencies |
| 29 | Ndensity(Stress-lemmas) | Orthographic neighbourhood - Coltheart's N based on the entries of the lexicon (with stress) |
| 30 | Ndensity(noStress-lemmas) | Orthographic neighbourhood - Coltheart's N based on the entries of the lexicon (ignoring stress) |
| 31 | Ndensity(Stress-wordfroms) | Orthographic neighbourhood - Coltheart's N based on the entries of the lexicon and their related  wordforms (with stress) |
| 32 | Ndensity(noStress-wordfroms) | Orthographic neighbourhood - Coltheart's N based on the entries of the lexicon and their related  wordforms (ignoring stress) |
| 33 | NdensityPhon(Stress-lemmas) | Phonological neighbourhood - Coltheart's N based on the entries of the lexicon (with stress) |
| 34 | NdensityPhon(noStress-lemmas) | Phonological neighbourhood - Coltheart's N based on the entries of the lexicon (ignoring stress) |
| 35 | NdensityPhon(Stress-wordfroms) | Phonological neighbourhood - Coltheart's N based on the entries of the lexicon and their related  wordforms (with stress) |
| 36 | NdensityPhon(noStress-wordfroms) | Phonological neighbourhood - Coltheart's N based on the entries of the lexicon and their related  wordforms (ignoring stress) |
| 37 | OLD20(stress-lemmas) | Levenshtein distance based on the orthographic entries of the lexicon (with stress) |
| 38 | OLD20(noStress-lemmas) | Levenshtein distance based on the orthographic entries of the lexicon (ignoring stress) |
| 39 | OLD20(stress-wordfroms) | Levenshtein distance based on the orthographic entries of the lexicon and their related wordforms (with stress) |
| 40 | OLD20(noStress-wordfroms) | Levenshtein distance based on the orthographic entries of the lexicon and their related wordforms (ignoring stress) |
| 41 | PLD20(stress-lemmas) | Levenshtein distance based on the phonological entries of the lexicon (with stress) |
| 42 | PLD20(noStress-lemmas) | Levenshtein distance based on the phonological entries of the lexicon (ignoring stress) |
| 43 | PLD20(stress-wordfroms) | Levenshtein distance based on the phonological entries of the lexicon and their related wordforms (with stress) |
| 44 | PLD20(noStress-wordfroms) | Levenshtein distance based on the phonological entries of the lexicon and their related wordforms (ignoring stress) |
| 45 | RhymeEnding(orth) | Orthographic string from the entry's stressed vowel up to the final letter of the word |
| 46 | RhymeN(orthLemmas-types) | Counts of lemmas matching the orthographic entry in orthographic rhyme ending |
| 47 | RhymeN(orthLemmas-tokens) | Standardized per million number of tokens matching the orthographic entry in orthographic rhyme  ending (only lemmas considered) |
| 48 | RhymeN(orthWordforms-types) | Counts of wordforms matching the orthographic entry in orthographic rhyme ending |
| 49 | RhymeN(orthWordforms-tokens) | Standardized per million number of tokens matching the orthographic entry in orthographic rhyme ending  (all wordforms considered) |
| 50 | RhymeEnding(phonol) | Phonological string from the entry's stressed vowel up to the final phone of the word |
| 51 | RhymeN(phonLemmas-types) | Counts of lemmas matching the phonological entry in phonological rhyme ending |
| 52 | RhymeN(phonLemmas-tokens) | Standardized per million number of tokens matching the phonological entry in phonological rhyme ending  (only lemmas considered) |
| 53 | RhymeN(phonWordforms-types) | Counts of wordforms matching the phonological entry in phonological rhyme ending |
| 54 | RhymeN(phonWordforms-types) | Standardized per million number of tokens matching the phonological entry in phonological rhyme ending  (all wordforms considered) |
| 55 | OrthEnding | Orthographic word ending comprising the nucleus vowel of the pre-final syllable and the whole final syllable |
| 56 | OrthEndingTypCount | Number of type counts of the word's orthographic ending in the database |
| 57 | EndingNconsistency(types) | Percentage of entries with the same orthographic ending stressed on the same syllable (based on type counts) |
| 58 | OrthEndingTokCount | Number of type counts of the word's orthographic ending in the database |
| 59 | OrthEndingNconsistency(tokens) | Percentage of entries with the same orthographic ending stressed on the same syllable (based on token counts) |
| 60 | PhonEnding | Phonological word ending comprising the nucleus vowel of the pre-final syllable and the whole final syllable |
| 61 | PhonEndingTypCount | Number of type counts of the word's phonological ending in the database |
| 62 | PhonEndingNconsistency(types) | Percentage of entries with the same phonological ending stressed on the same syllable (based on type counts) |
| 63 | PhonEndingTokCount | Number of type counts of the word's phonological ending in the database |
| 64 | PhonEndingNconsistency(tokens) | Percentage of entries with the same phonological ending stressed on the same syllable (based on token counts) |
| 65 | POS | Part of Speech category |

*Hellenic National Corpus

**Table 2. Stress Neighbourhood statistics.**

| Column | Name | Property |
| --- | --- | --- |
| 1 | ending | Each unique ending that occurs in the database |
| 2 | count | Number of counts it occurs |
| 3 | mono | Number of times it is observed in a monosyllabic entry |
| 4 | APU | Number of times it is observed in a polysyllabic entry stressed on the antepenultimate |
| 5 | PU | Number of times it is observed in a polysyllabic entry stressed on the pre-final |
| 6 | U | Number of times it is observed in a polysyllabic entry stressed on the final |

Four such files are provided, for the phonological and orthographic forms as well as type and token frequencies

**Table 3. Syllabic units information.**

| Column | Name | Property |
| --- | --- | --- |
| 1 | Syllable | Each unique syllabic unit that occurs in the database |
| 2 | TypeCounts | Number of counts it occurs in the database's types |
| 3 | TokenCounts | Number of counts it occurs in the database's tokens |
| 4 | TypeFreq | Frequency per million of occurrence in the database's types |
| 5 | TokenFreq | Frequency per million of occurrence in the database's tokens |

Eight such files are provided, for the phonological and orthographic forms, considering or ignoring stress diacritics, as well as considering lemmas only or all wordforms.

**Table 4. Part-of-speech information.**

| Column | Name | Property |
| --- | --- | --- |
| 1 | PosTag | Each PoS tag's abbreviation as it appears in the database file |
| 2 | PosCateg | PoS category each tag corresponds to |
| 3 | Counts | Number of counts each PoS category occrus in the database |
| 4 | PosFreq (%) | Frequency per cent of each PoS category's occurrence in the database |

**Table 5. Comparison of syllabic length vs. stress position between GreekLex 2 and IPLR.**

| Column | Name | Property |
| --- | --- | --- |
| 1 | sylLength | The syllabic length of each clustering category |
| 2 | StressedSyllable | The stress position of each clustering category |
| 3 | Counts | The type of measurement reported (types vs. tokens) |
| 4 | GreekLex 2 | The number of counts in GreekLex 2 |
| 5 | IPLR | The number of counts in IPLR |

**Table 6. Comparison of the inconsistent phonetic transcriptions between GreekLex 2 and IPLR.**

| Column | Name | Property |
| --- | --- | --- |
| 1 | GreekLex 2 | The phonetic transcription in GreekLex 2 |
| 2 | IPLR | The phonetic transcription in IPLR |
